# Disparities of Mortality Trends Due to Cerebrovascular Diseases and Cerebrovascular Infarction in the United States

**DOI:** 10.1161/SVIN.123.001158

**Published:** 2024-05-21

**Authors:** Sishir Doddi, Nicholas D. Henkel, Oscar Salichs, Richard Burgess, Taryn Hibshman, Jonathan Wright, Isa Malik, Sami Al Kasab, Mouhammad A. Jumaa

**Affiliations:** ^1^ University of Toledo College of Medicine Toledo OH; ^2^ Department of Neurology ProMedica Neurosciences Institute Toledo OH; ^3^ Department of Neurology University of Toledo College of Medicine Toledo OH; ^4^ College of Medicine The Ohio State University Columbus OH; ^5^ Medical University of South Carolina Charleston SC

**Keywords:** demographics, disparities, mortality, stroke

## Abstract

**Background:**

Cerebrovascular diseases are a major cause of morbidity and mortality worldwide and are the fifth leading cause of death in the United States. Understanding demographic differences in mortality rate trends can raise awareness of demographic disparities. We sought to investigate age‐adjusted mortality trends due to cerebrovascular diseases and ischemic stroke for demographic disparities in trend from 1999 to 2020.

**Methods:**

We used the publicly accessible Centers for Disease Control and Prevention Wide‐Ranging Online Data for Epidemiologic Research database to gather mortality data to determine trends in cerebrovascular diseases and cerebral infarction mortality in the United States from 1999 to 2020. Using the Joinpoint program, temporal trends for cerebrovascular diseases and cerebral infarction mortality were calculated for each demographic group and reported as both annual percentage changes (APCs) or average APC from 1999 to 2020. In addition, trends were compared between groups for significant differences.

**Results:**

We found an overall decrease in mortality rate for cerebrovascular diseases with average APC −1.9%. In 2020, age‐adjusted mortality rates due to cerebrovascular diseases in the Black population was 1031 per 1 000 000 compared with 679 in the White population. Similarly in 2020, cerebral infarction for the Black population had an age‐adjusted mortality rate of 256.3 compared with the White population's 170.4. When assessing overall trends by race and ethnic group: American Indian/Alaska Native had average APC −2.5%, Asian Pacific Americans had 2.4%, White population had −1.9%, and the Black population had −1.8%. We found a statistically significant difference in trend of decline between the Black and White population cerebrovascular diseases age‐adjusted mortality rates. No significant average APCs were found for cerebral infarction. The results of this study showcase disparities in cerebrovascular diseases mortality in the United States and where additional effort, research, and care should be focused.

**Conclusion:**

The results of this study showcase disparities in mortality in the United States and where additional effort, research, and care should be focused.

**Nonstandard Abbreviations and Acronyms**

AAMR: age‐adjusted mortality rate
AAPC: average annual percentage change
AAPI: Asian or Pacific Islander
AI/AN: American Indian or Alaskan Native
APC: annual percentage change
CDC: Centers for Disease Control and Prevention
MT: mechanical thrombectomy
WONDER: Wide‐Ranging Online Data for Epidemiologic Research

Stroke is a major cause of morbidity and mortality worldwide and is the fifth leading cause of death in the United States.^1^ Stroke is often categorized as either hemorrhagic or ischemic. Hemorrhagic stroke is most often secondary to hypertensive rupture of microaneurysms in penetrating vessels, saccular aneurysms, or arteriovenous malformations. Ischemic stroke accounts for 87% of all strokes and is typically secondary to occlusion of blood vessels supplying the brain, most often embolic or thrombotic. Either result in ischemia of the central nervous system, causing damage and possibly death. The incidence of cerebrovascular disease is expected to increase due to an aging population and increasing prevalence of risk factors, such as hypertension, diabetes, and obesity.^2^ Despite advancements in cerebrovascular disease management and prevention over the past 2 decades, cerebrovascular disease remains a significant burden on the US health care system.

In this study, we assess stroke‐related morbidity and mortality trends stratified by race and sex using the Centers for Disease Control and Prevention (CDC) Wide‐Ranging Online Data for Epidemiologic Research (WONDER) database. Given information on cerebrovascular disease disparities from previous studies,^3^, ^4^ we hypothesize there will be disparities in our primary end point, age‐adjusted mortality rate (AAMR) reduction trend, by race categories and sex from 1999 to 2020 for cerebrovascular diseases and cerebral infarction.

## Methods

Data can be made available upon request. The public CDC WONDER was used to access multiple cause of death mortality data with *International Classification of Diseases, Tenth Revision* (*ICD‐10*) codes: I60 to I69 (all‐cause cerebrovascular disease) and I63 (cerebral infarction) from 1999 to 2020. The CDC WONDER uses death certificates to gather data on cause of death, place of death, and demographic information. The data set was queried to gather mortality information due to cerebrovascular disease of various demographic groups from 1999 to 2020. The AAMRs with either cerebrovascular disease or cerebral infarction as an underlying cause of death, were collected and separated by sex and race. For purpose of this study, the categories for race are: American Indian or Alaska Native (AI/AN), Asian or Pacific Islander (AAPI), Black or African American, and White. Institutional review board approval was not required as CDC WONDER uses public deidentified information. Ethnicity refers to whether a person is of Hispanic origin or not.

The CDC WONDER database calculated the age‐adjusted mortality rate per 1 000 000 and associated SE values for mortality due to cerebrovascular infarction in the 1999 to 2020 time period. AAMR is calculated by mortality number/total population and standardizing the value to the 2000 US standard population. Using the Joinpoint Regression Program (Joinpoint V 4.9.0.0, National Cancer Institute), significant trends in mortality rates for each group were calculated by determining the annual percentage change (APC) for each year between 1999 and 2020.^5^ Additionally, significant average APC (AAPC) from 1999 to 2020 was calculated. The program analyzed APC trends for significance using log‐linear regression models, which has been previously used to assess significant trends in recent literature.^6^, ^7^, ^8^ APCs and corresponding 95% CIs were calculated using the Grid Search Method (2,2,0), permutation, and parametric test. Significant differences in trend between groups were calculated using a parallel pairwise comparison, which compares segmented line regression models.^9^ Significance for all tests is set at *P* < 0.05. The methods and results of this study adhere to the EQUATOR Strenghtening the Reporting of Observational Studies in Epidemiology (STROBE) reporting guidelines.^10^

## Results

### Total Population Cerebrovascular Disease

**Figure 1:**
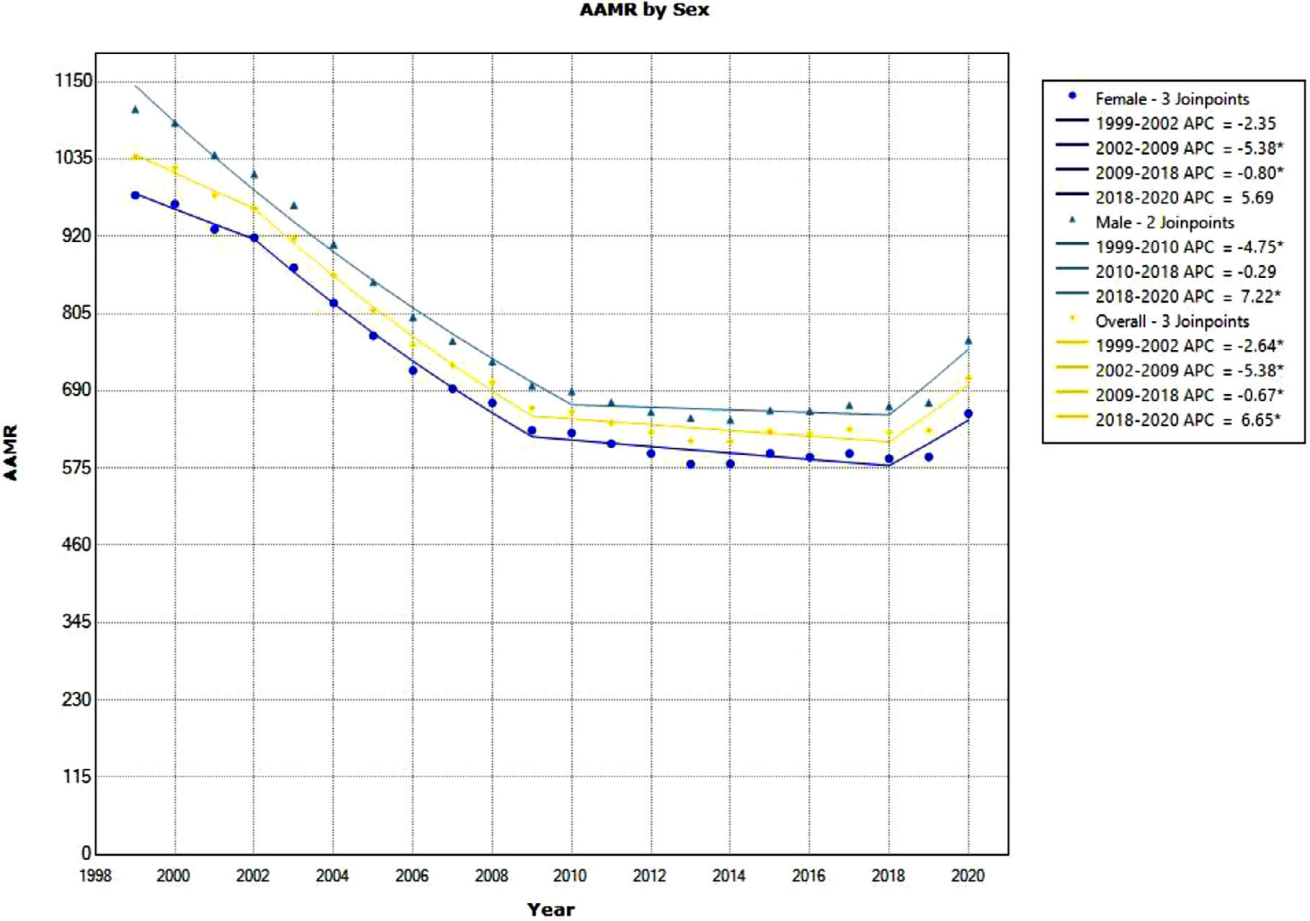
**Age‐adjusted mortality rate (AAMR) due to cerebrovascular disease between 1999 and 2020 by sex**. The graph depicts AAMR per 1 000 000 between 1999 and 2020 for men, women, and the total US population. Significant trends are annual percentage change (APC) values with an asterisk. CIs of APC are provided in the supplement.

From 1999 to 2020, a total of 5 387 782 events of mortality occurred with cerebrovascular disease (*ICD‐10*: I60–I69 [cerebrovascular disease]) as a contributing cause of death. The overall population's AAMR due to all‐cause cerebrovascular disease in 1999 was 1038.0. This value has decreased to 709.1 in 2020. Four significant trends were found for the overall US population in this period, 3 downtrends and 1 uptrend. From 1999 to 2002, there was a downtrend with APC −2.64%, 2002 to 2009 with APC −5.38, and 2009 to 2018 with APC −0.67%. From 2018 to 2020, there was an uptrend with APC of 6.65%. The overall AAPC from 1999 to 2020 was −1.9% (Table 1, Figure 1).

**Table 1:** Total Population Cerebrovascular Disease and Cerebrovascular Infarction

Year	AAPC, %	95% CI, %	*P* value
**Cerebrovascular diseases**			
1999–2020	−1.90	−2.5 to −1.2	<0.001

Year	APC, %	95% CI, %	*P* value
1999–2002	−2.64	−5.1 to −0.1	0.04
2002–2009	−5.38	−6.2 to −4.5	<0.001
2009–2018	−0.67	−1.3 to −0.1	0.32
2018–2020	6.65	1.3 to 12.2	<0.001
**Cerebrovascular infarction**			
1999–2003	−6.44	−10.8 to −1.8	0.013
2003–2006	−18.67	−33.2 to −1.0	0.042
2006–2014	−3.16	−6.2 to −0.2	0.039
2014–2017	31.22	7.8 to 59.7	0.013

AAPC indicates average APC; and APC, annual percentage change.

### Total Population Cerebrovascular Infarction

**Figure 2:**
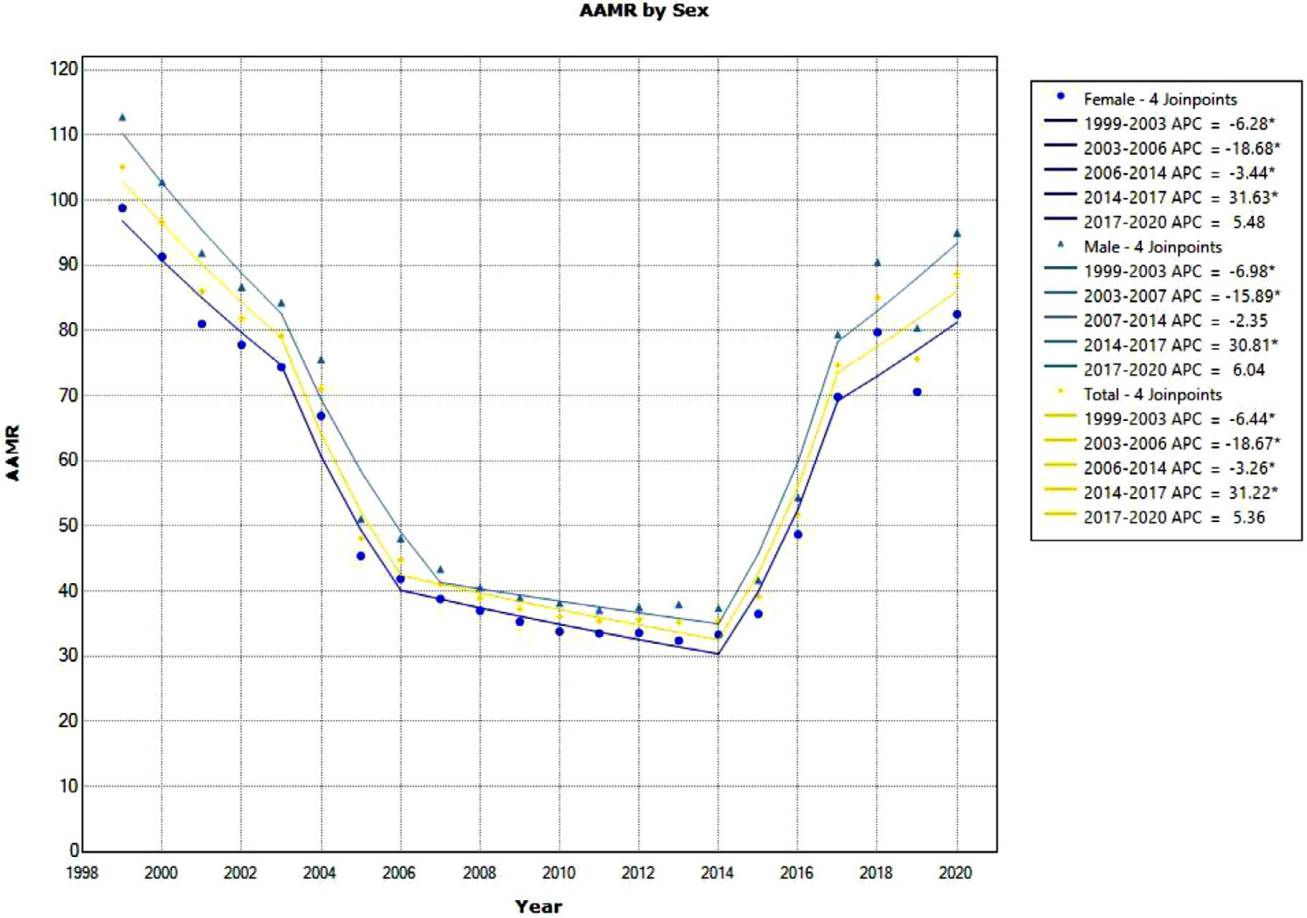
**Age‐adjusted mortality rate (AAMR) due to cerebrovascular infarction between 1999 and 2020 by sex**. The graph depicts AAMR per 1 000 000 between 1999 and 2020 for men, women, and the total US population. Significant trends are annual percentage change (APC) values with an asterisk. CIs of APC are provided in the supplement.

In the 1999 to 2020 time period, cerebrovascular infarction (*ICD‐10* code: I63 [cerebral infarction]) represents 438 190 causes of death in the United States. In 1999, the AAMR was 105.1 per 1 000 000, and in 2020, it was 88.7, showing an overall decrease in mortality rate (Figure 2). During this time period, 4 significant trends were observed for the overall population: 1999 to 2003 had an APC of −6.44%, 2003 to 2006 had an APC of −18.67%, 2006 to 2014 had an APC of −3.16%, and 2014 to 2017 had an APC of 31.22%. No significant AAPC from 1999 to 2020 was found for the total population (Table 1, Figure 2).

### Cerebrovascular Disease by Sex

For all‐cause cerebrovascular disease, in 1999, men had an AAMR of 1108.9 per 1 000 000, and in 2020, they had an AAMR of 765.6. The male population observed 1 downtrend, 1999 to 2010 with APC −4.75%, and 1 uptrend, 2018 to 2020 with APC 7.2%. In 1999, women had an AAMR of 981.0, and in 2020, they had an AAMR of 656.5 (Figure 1). The AAPC in the overall time period for men was −2.0%.

Typically, for a given year, women had a lower AAMR due to cerebrovascular disease when compared with men. In this period, women observed 2 downtrends: 1999 to 2010 APC −5.38% and 2009 to 2018 APC −0.80%. Similar to men, the AAPC in the overall time period for women was −2.0%. Pairwise parallelism test found both men and women to have similar trends (Table 2, Figure 1).

**Table 2:** Cerebrovascular Disease and Cerebrovascular Infarction Rates by Sex

Cerebrovascular disease				
Sex	AAPC, %	Year	95% CI, %	*P* value
Female	−2.00	1999–2020	−2.6 to −1.3	<0.001
Male	−2.00	1999–2020	−2.5 to −1.5	<0.001

Sex	APC, %	Year	95% CI, %	*P* value
Female	−5.38	1999–2010	−6.3 to −4.5	<0.001
Female	−0.80	2009–2018	−1.4 to −0.2	0.019
Male	−4.75	1999–2010	−5.1 to −4.4	<0.001
Male	7.20	2018–2020	2.2 to 12.5	0.007
**Cerebrovascular infarction**				
Female	−6.28	1999–2003	−10.5 to −1.8	0.012
Female	−18.68	2003–2006	−31.6 to −3.3	0.025
Female	−3.44	2006–2014	−6.1 to −0.7	0.02
Female	31.63	2014–2017	−2.5 to −1.5	0.009
Male	−6.98	1999–2003	−12.9 to −0.7	0.034
Male	−15.89	2003–2007	−25.3 to −5.3	0.01
Male	30.81	2014–2017	3.2 to 65.8	0.031

AAPC indicates average APC; and APC, annual percentage change.

### Cerebrovascular Infarction by Sex

For cerebrovascular infarction, in 1999, men had an AAMR of 112.8 per 1 000 000, which decreased to 95.0 in 2020. Men had 3 significant downtrends from 1999 to 2020. A decrease was found from 1999 to 2003, APC −6.98%, 2003 to 2007, APC −15.89%, and 2014 to 2017, APC 30.81%. Compared with men, women had lower AAMRs. In 1999, women had an AAMR due to cerebrovascular infarction of 98.8, and that of 82.5 in 2020. No significant AAPC from 1999 to 2020 was found for the male population (Table 2).

In the time period of this study, women observed 4 significant trends: 1999 to 2003 APC −6.28%, 2003 to 2006 APC −18.68%, 2006 to 2014 APC −3.44%, and 2014 to 2017 APC 31.63%. No significant AAPC from 1999 to 2020 was found for the female population (Table 2, Figure 2).

### Cerebrovascular Disease by Race

**Figure 3:**
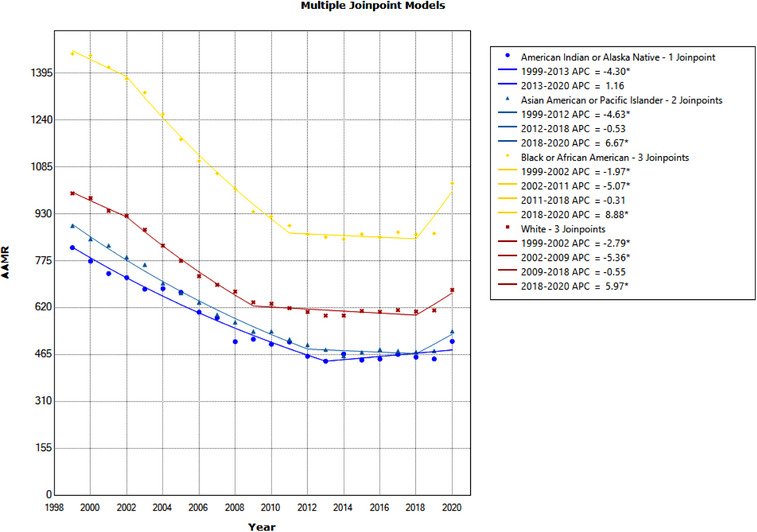
**Age‐adjusted mortality rate (AAMR) due to cerebrovascular disease between 1999 and 2020 by race and ethnicity**. AAMR per 1 000 000 between 1999 and 2020 separated by race: American Indian or Alaskan Native, Asian or Pacific Islander, Black or African American, and White. Significant trends are annual percentage change (APC) values with an asterisk.

For all cerebrovascular diseases, AAMRs differed by race and ethnic group. In 1999, the AAMR for the Black population was the highest among groups at 1458.2 per 1 000 000. In the same year, the White population had an AAMR of 997.4, AAPI had an AAMR of 890.4, and the AI/AN population had an AAMR of 818.1. In 2020, these values decreased for all groups. The Black, White, AAPI, and AI/AN population AAMRs in 2020 were 1030.7, 678.7, 542.3, and 508.5, respectively (Figure 3). When assessing overall AAPC trends, the AI/AN group had the highest AAPC with −2.5%, followed by the AAPI population with AAPC −2.4%, the White population with AAPC −1.9%, and the Black population with AAPC −1.8%. Pairwise parallelism tests between groups found a significant difference in trend between the Black and White populations, where the rate of decline in cerebrovascular disease mortality was significantly lower in the Black population when compared with the White population from 1999 to 2020 (*P* = 0.0016) (Table 3).

**Table 3:** Cerebrovascular Disease and Cerebrovascular Infarction by Race

Race	AAPC, %	Year	95% CI, %	*P* value
AAPI	−2.40	1999–2020	−3.0 to −1.9	<0.001
Black	1.80	1999–2020	−2.2 to −1.4	<0.001
AI/AN	−2.50	1999–2020	−3.1 to −2.0	<0.001
White	−1.90	1999–2020	−2.5 to −1.3	<0.001

Race	APC, %	Year	95% CI, %	*P* value
AAPI	−4.60	1999–2012	−4.9 to −4.3	<0.001
AAPI	6.70	2018–2020	2.1 to 11.5	0.007
Black	−2.00	1999–2002	−3.7 to −0.2	0.031
Black	−5.10	2002–2011	−5.5 to −4.7	<0.001
Black	8.90	2018–2020	5.3 to 12.6	<0.001
AI/AN	−4.30	1999–2013	−4.9 to −3.7	<0.001
White	−2.80	1999–2002	−5.1 to −0.4	0.025
White	−5.40	2002–2009	−6.2 to −4.5	<0.001
White	6	2018–2020	0.8 to 11.4	0.027
**Cerebrovascular infarction**				
AAPI	−12.47	1999–2008	−15.9 to −8.9	<0.001
Black	−10.24	1999–2012	−12.1 to −8.3	<0.001
Black	17.10	2012–2020	12.7 to 21.7	<0.001
AI/AN	−8.96	1999–2012	−11.3 to −6.6	<0.001
AI/AN	15.95	2012–2020	11.5 to 20.5	<0.001
White	−10.95	1999–2008	−13.2 to −8.6	<0.001

AAPC indicates average APC; AAPI, Asian or Pacific Islander; AI/AN, American Indian or Alaskan Native; and APC, annual percentage change.

In the time period of this study, the Black population had 3 significant trends: 2 downtrends and 1 uptrend. From 1999 to 2002, there was a downtrend of APC −2.0% and 2002 to 2011 with APC −5.1%. From 2018 to 2020, there was an uptrend with APC 8.9%. In this time period, the White population also had 3 trends: from 1999 to 2002, there was an observed APC of −2.8%, 2002 to 2009 had APC of −5.4%, and 2018 to 2020 had APC of 6%. The AAPI population had 2 significant trends: 1999 to 2012, APC −4.6%; and 2018 to 2020, APC 6.7%. The AI/AN population had 1 significant trend from 1999 to 2013 with APC of −4.3% (Table 3, Figure 3).

### Cerebrovascular Infarction by Race

**Figure 4:**
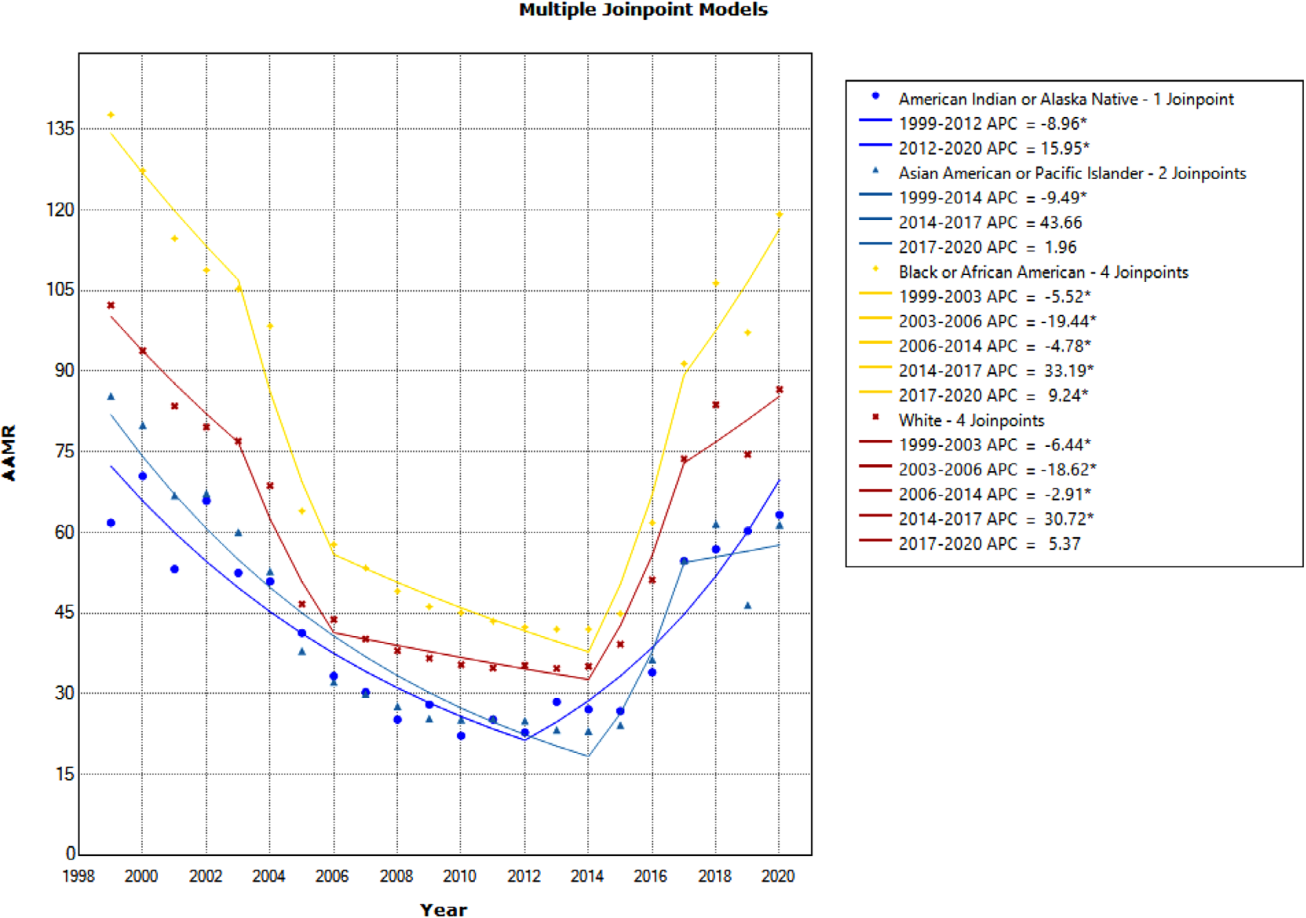
**Age‐adjusted mortality rate (AAMR) due to cerebrovascular infarction between 1999 and 2020 by race and ethnicity**. AAMR per 1 000 000 between 1999 and 2020 separated by race: American Indian or Alaskan Native, Asian or Pacific Islander, Black or African American, and White. Significant trends are annual percentage change (APC) values with an asterisk.

When assessing AAMR for cerebrovascular infarction by race and ethnic group, differences based on group were found. In 1999, the AAMR for the Black population was the highest at 137.7 per 1 000 000. The group with the second highest was the White population with AAMR of 102.3, followed by the AAPI and AI/AN populations (85.4 and 61.8, respectively). Compared with 1999, in 2020, the AAMR due to cerebrovascular infarction decreased for all groups (Figure 4). No statistically significant AAPC trend from 1999 to 2020 for any race and ethnic group could be calculated. In 2020, the AAMRs for the Black, White, AAPI, and AI/AN populations were 119.2, 86.6, 61.5, and 63.3, respectively. Two significant downtrends were found in the Black population. From 1999 to 2012, the APC was −10.24%, and from 2012 to 2020, the APC was 17.1%. The White population had only 1 significant trend, between 1999 and 2008, the APC was −10.95%. The AAPI population also had 1 significant trend between 1999 and 2008, APC −12.47%. The AI/AN group had 2 significant trends, one 1999 to 2012 with APC −8.96%, and 2012 to 2020 with APC 15.95%. In a pairwise test for parallelism to compare trends, no significant difference in trends was found between any groups (Table 3, Figure 4).

### Cerebrovascular Disease by Ethnicity

In 1999, the non‐Hispanic population had an AAMR of 1043 due to all‐cause cerebrovascular disease and the Hispanic population had an AAMR of 828. In 2020, the non‐Hispanic population's AAMR decreased to 716 and the Hispanic population's AAMR decreased to 626. The AAPC from 1999 to 2020 for the non‐Hispanic population was −1.9%, and for the Hispanic population, it was −1.6%. Parallel pairwise comparison found no significant difference in trend between the 2 groups. When investigating temporal trends, the non‐Hispanic population had 4 significant trends: from 1999 to 2002, APC −2.6%, 2002 to 2009, APC −5.3%, 2009 to 2018, APC −0.6%, and 2018 to 2020, APC 6.4%. The Hispanic population observed 2 significant trends. From 1999 to 2011, a downtrend of APC −4.1% was observed, and from 2018 to 2020, an uptrend of APC 9% was observed (Table 4).

**Table 4:** Cerebrovascular Disease by Ethnicity

Ethnicity	AAPC, %	Year	95% CI, %	*P* value
Hispanic	−1.60	1999–2020	−2.1 to −1.1	<0.001
Non‐Hispanic	−1.90	1999–2020	−2.5 to −1.2	<0.001

Ethnicity	APC, %	Year	95% CI, %	*P* value
Hispanic	−4.10	1999–2011	−4.4 to −3.7	<0.001
Hispanic	9	2018–2020	4.3 to 13.9	0.001
Non‐Hispanic	−2.60	1999–2002	−4.9 to −0.2	0.036
Non‐Hispanic	−5.30	2002–2009	−6.2 to −4.5	<0.001
Non‐Hispanic	−0.60	2009–2018	−1.2 to 0	0.047
Non‐Hispanic	6.40	2018–2020	1.1 to 11.9	0.021

AAPC indicates average APC; and APC, annual percentage change.

### Cerebrovascular Infarction by Ethnicity

In 1999, the AAMR due to cerebrovascular infarction for the Hispanic population was 732 per 1 000 000 and 1062 for the non‐Hispanic population. In 2020, the rate increased to 864 for the Hispanic population and increased to 889 for the non‐Hispanic population. No statistically significant AAPC from 1999 to 2020 was found for the Hispanic or non‐Hispanic populations; however, significant temporal trends were found. The non‐Hispanic population had 4 significant temporal trends from 1999 to 2020: from 1999 to 2003, there was a decline, APC −6.2% (95% CI, −8.2% to −4.2%; *P*<0.001); from 2003 to 2006, there was a decline, APC −18.9%; 2006 to 2014 had an APC −2.9%; and an uptrend was found from 2014 to 2018, APC 25.9%. The Hispanic population observed 2 significant trends: from 1999 to 2008, a downtrend was observed, APC −11.1%; and from 2014 to 2017, an uptrend was observed, APC 37.8%. Parallel pairwise comparison found no significant difference in trend between the 2 ethnic groups (Table 5).

**Table 5:** Cerebrovascular Infarction by Ethnicity

Ethnicity	APC, %	Year	95% CI, %	*P* value
Hispanic	−11.10	1999–2008	−13.8 to −8.3	<0.001
Hispanic	37.80	2014–2017	7.3 to 77.0	0.017
Non‐Hispanic	−6.20	1999–2003	−8.2 to −4.2	<0.001
Non‐Hispanic	−18.90	2003–2006	−25.1 to −12.1	<0.001
Non‐Hispanic	−2.90	2006–2014	−4.1 to −1.6	0.001
Non‐Hispanic	25.90	2014–2018	21.3 to 30	<0.0017

APC indicates annual percentage change.

### Place of Death

**Figure 5:**
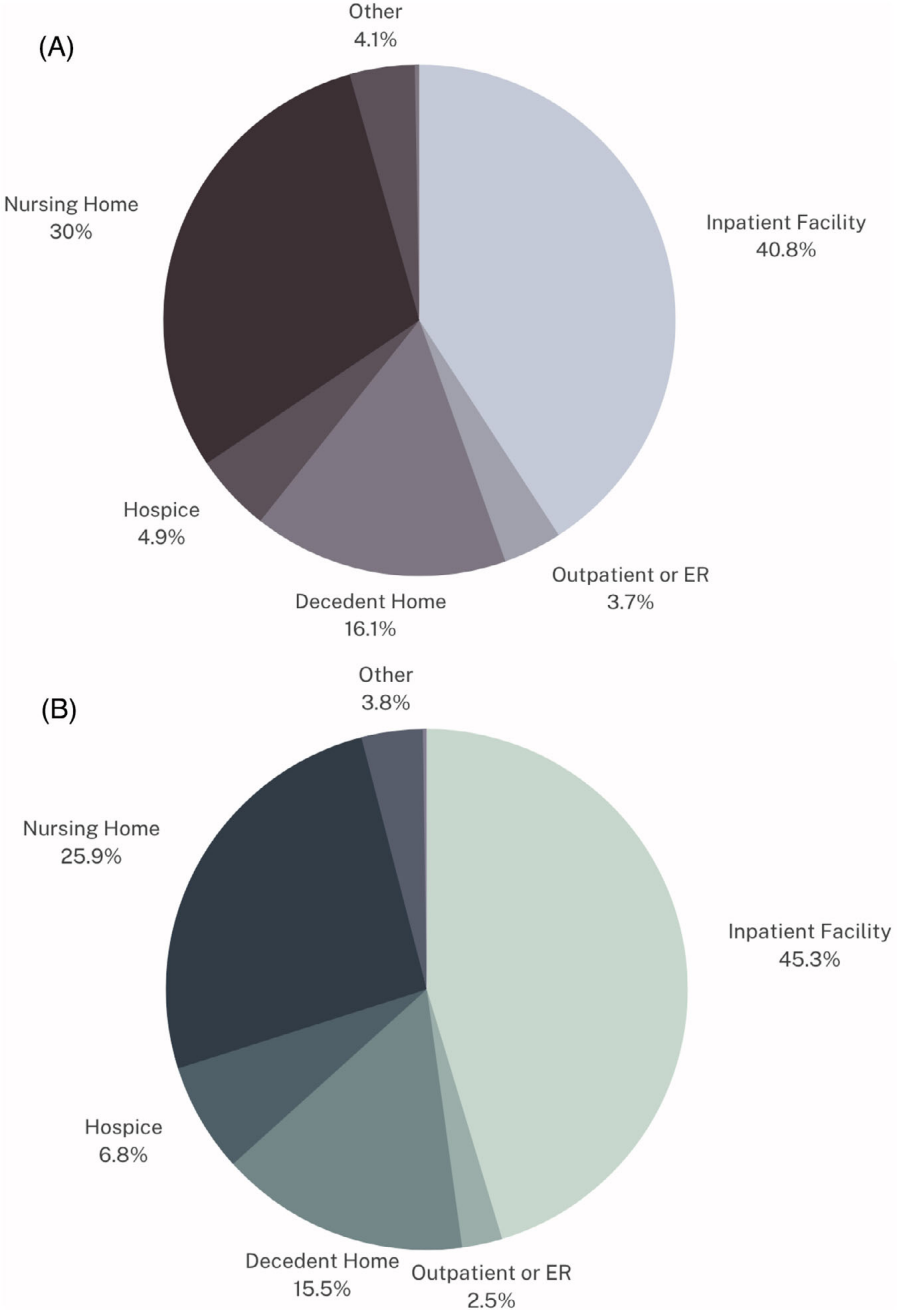
**Location of mortality due to cerebrovascular disease and ischemic stroke. A**, Places of death due to stroke in the United States 1999 to 2020. This figure depicts the most common locations of death for individuals with *International Classification of Diseases, Tenth Revision* (*ICD‐10*) codes I60 to I69 as a multiple cause of death. **B**, Places of death due to cerebrovascular infarction in the United States 1999 to 2020. This figure depicts the most common locations of death for individuals with *ICD‐10* code I63 as a multiple cause of death. ER indicates emergency department.

Most events of mortality occurred in inpatient medical facilities, followed by in nursing homes, home of a descendent, hospice facilities, emergency department or outpatient setting, other/unknown status, and dead on arrival (Figure 5A). The same ranking for most common place of death was observed for cerebrovascular infarction (Figure 5B).

## Discussion

To our knowledge, we provide the first report investigating mortality trends by demographic groups in the United States for both all‐cause cerebrovascular disease (*ICD‐10* codes: I60–I69) and cerebrovascular infarction (*ICD‐10* code: I63). With the CDC WONDER database, we extracted differential AAMRs and mortality trends for various demographic cohorts from 1999 to 2020.

Mortality with all‐cause cerebrovascular disease (*ICD‐10*: I60–I69 [cerebrovascular disease]) and cerebral infarction decreased from 1999 to 2020 in the United States. Declining mortality rates were not limited to the United States: decreased stroke mortality has been reported in China.^11^ Globally, stroke mortality rates (from 1990 to 2010) have declined.^12^ In general, as age group is increased, AAMR due to strokes increases as well.^13^

In our analysis, men and women display similar temporal trends of cerebrovascular disease and cerebrovascular infarction mortality. However, previous reports show conflicting data on stroke‐induced hospitalization. Men, specifically those aged between 45 and 64 years, have higher hospitalization rates,^14^ despite women having a higher risk of thrombotic ischemic stroke.^15^ Such outcome measures may be age dependent. Women have poorer outcomes when aged >65 years compared with men of the same age, but, if aged <65 years, mortality outcomes do not significantly differ.^16^ Moreover, women are less likely to receive interventional therapy, such as tPA (tissue‐type plasminogen activator) therapy, compared with men.^17^, ^18^

All observed race and ethnic groups have decreased cerebrovascular disease rates from 1999 to 2020. AAMRs, alternatively, differed by group. In 2020, the Black population had the highest AAMR, followed by the White, AI/AN, and AAPI populations. When assessing mortality rates by race and ethnic group for all‐cause cerebrovascular disease, we report differential temporal trends. Previously, stroke incidence disproportionately increased in the Black population compared with the remaining US population.^19^ Similarly, compared with the White population, the Black population significantly differed in AAMR trend in our analysis (Supplement 3). These findings indicate unfortunate racial disparities in cerebrovascular outcomes.

Similar to our results for cerebrovascular disease events, our analysis shows declining trends for all race and ethnic groups due to cerebrovascular infarction. However, AAMRs differ by group. In 2020, the Black population had the highest AAMR due to cerebrovascular infarction, followed by the White population, and then the AI/AN and AAPI populations (Figure 2). A notable observation in 2020 is that the Black AAMR is double that of the AI/AN and AAPI groups. Thrombotic events are disproportionately more likely to happen in the Black population and are less likely to happen in the Asian American population.^20^ Socioeconomic factors may in part explain the high cerebrovascular infarction AAMR for the Black population and low rate for the AAPI group. Furthermore, the Black population is at a greater risk of cerebrovascular infarction recurrence.^21^ Furthermore, compared with the White population, the Black population is less likely to receive reperfusion therapy, intravenous thrombolytics, and mechanical thrombectomy (MT).^22^, ^23^, ^24^ Furthermore, socioeconomic factors, including poverty, insurance status, restricted access to health care facilities and resources, and lack of proper patient education, contribute to increased stroke mortality rates.^25^ Addressing these systemic issues is integral in improving mortality rates and disparities in cerebrovascular infarction.

Several developments and advances in care for cerebrovascular disease and cerebrovascular infarction likely contributed to the overall decrease in mortality since 1999. Improving patient cerebrovascular disease outcomes relies on reducing the time between cerebrovascular disease onset and delivery of medical care. Since 1999, there have been an increased number of established stroke care centers.^26^ These specialized centers have health professionals dedicated to stroke evaluation and treatment.^27^ Patients with stroke subsequently have access to stroke‐specific care, effectively minimizing cerebrovascular infarction mortality.^28^ A previous study determined that increasing connections between hospitals and decreased patient transfer times are associated with improved patient outcomes for stroke.^29^ Telemedicine also reduces time to deliver stroke‐specific care for patients with restricted or limited access to receive quality evaluation and care by allowing for patients in mobile medical stroke units to be evaluated by a specialists while in transit.^30^ To improve quality of life after a stroke event, remote telerehabilitation can assist recovering patients.^31^

A major factor contributing to the declining rate of cerebrovascular infarction mortality since 1999 was the widespread use of tPA or alteplase. Although tPA was first US Food and Drug Administration approved in 1996, its use did not become common until following years.^32^ Further advancements led to increased benefit from recombinant tPA, advancements in imaging, intravascular use of recombinant tPA, and combination of tPA with other therapeutics.^32^, ^33^ A second development in cerebrovascular infarction care is MT. MT improves functional independence and reduces mortality compared with medical therapy alone.^34^, ^35^ MT, in combination with fibrinolytic therapy, is the cornerstone of stroke‐reperfusion therapy. Multiple clinical trials have sought to improve fibrinolytic therapy with modified tPA. Tenecteplase, which differs from alteplase by 3 amino acids, has a higher fibrin specificity than its counterpart. For this reason, it has been hypothesized to have higher efficacy in reperfusion and functional outcomes in endovascular therapy. To date, however, the evidence for higher efficacy is equivocal.^36^, ^37^, ^38^, ^39^, ^40^, ^41^ Together, advancements in interventional neurology have drastically improved stroke outcomes since the early 2000s. The advent of intravenous reperfusion therapy in the late 1990s and accelerating use of MT between 2013 and 2020 have been significant drivers of mortality gains.

A notable cause for decreased stroke mortality is the increased use and efficacy of prevention guidelines. These guidelines aim to improve public understanding of health and decrease stroke risk factors, mainly hypertension, diabetes, and hypercholesterolemia. The relative health improvement and mitigation of stroke risk factors likely contributed to the overall decrease in stroke and cerebral infarction mortality.^42^, ^43^, ^44^ Hypertension incidence has decreased in the United States since 2013.^45^ From 1999 to 2010, the diabetic US population had improved glycemic control; however, since 2015, glycemic control decreased.^46^ Cholesterol management in US adults increased from 1999 to 2010, yet subsequently plateaued.^46^

This study has flaws, and its results should be considered with these limitations. The CDC WONDER database is composed of data extracted from death certificates between 1999 and 2020. Causes of death are listed on death certificates only when cerebrovascular disease or cerebral infarction is diagnosed during intervention or at autopsy at a medical center. Therefore, individuals who lack access to proper medical treatment may not have cerebrovascular disease or cerebrovascular infarction listed on the certificate. In addition, before the widespread implementation of electronic medical records, diagnoses could be misclassified due to limited available medical history. Finally, there may be classification errors when data are inputted into the database.

In conclusion, from 1999 to 2020, mortality rates have declined for both cerebrovascular disease and specifically for cerebrovascular infarction in the United States. In 2020, there were similar rates of age‐adjusted all‐cause mortality for both men and women. All‐cause mortality rate trends were also similar for race and ethnic groups. However, disparities in AAMR and AAPC by race and ethnic groups were found in cerebrovascular diseases. Specifically, the Black population had a higher rate of mortality compared with other ethnic groups for cerebrovascular disease and cerebral infarction. Additionally, it was found that the rate of decline in stroke mortality in the Black population lagged compared with the White population from 1999 to 2020. The results of this study showcase disparities in cerebrovascular‐related mortality in the United States, and it is toward addressing these disparities that additional effort, research, and care should be focused.

## Author Contributions

Sishir Doddi: conceptualization, methods, software, data curation, writing (original draft preparation), visualization, and investigation. Nicholas D. Henkel, Richard Burgess, Oscar Salichs, Taryn Hibshman, Jonathan Wright, and Isa Malik: writing, reviewing, and editing. Sami Al Kasab: reviewing and editing. Mouhammad A. Jumaa: reviewing and editing and supervision.

## Disclosures

Sami Al Kasab serves on the Editorial Board of S:VIN. Editorial Board Members are not involved in the handling or final disposition of submissions.
